# Supplementary material for: Gender-based violence and depressive symptoms among female entertainment workers in Cambodia: A cross-sectional study
Source: PLOS Glob Public Health. 2022 Aug 3;2(8):e0000873. doi: 10.1371/journal.pgph.0000873 (PMC10021637; doi:10.1371/journal.pgph.0000873)
Supplement: S1 Table — (DOCX) [file pgph.0000873.s001.docx]

| **Table 1S. Comparison of socio-demographic characteristics of FEWs with and without experiencing each type of GBV and at least one type of GBV (n=645).** | | | | | | | | | | | | | | | | | | | | |  |
| --- | --- | --- | --- | --- | --- | --- | --- | --- | --- | --- | --- | --- | --- | --- | --- | --- | --- | --- | --- | --- | --- |
| **Variable** | **Physical violence*** | | | | | **Sexual violence*** | | | | | | | **Emotional abuse*** | | | | **At least one type of GBV*** | | | | |
|  | **No** | | **Yes** | ***p*-value^†^** | **No** | | | **Yes** | | ***p*-value^†^** | | **No** | | **Yes** | ***p*-value^†^** | **No** | | **Yes** | ***p*-value^†^** |  |  |
|  | **N (%)** | | **N (%)** |  | **N (%)** | | | **N (%)** | |  | | **N (%)** | | **N (%)** |  | **N (%)** | | **N (%)** |  |  |  |
| Study site |  | |  | 0.186 |  | | |  | | 0.037 | |  | |  | <0.001 |  | |  | <0.001 |  |  |
| Phnom Penh | 225 (84.3) | | 42 (15.7) |  | 210 (78.6) | | | 57 (21.4) | |  | | 122 (45.7) | | 145 (54.3) |  | 107 (40.1) | | 160 (59.9) |  |  |  |
| Pursat | 65 (92.9) | | 5 (7.1) |  | 59 (84.3) | | | 11 (15.7) | |  | | 49 (70.0) | | 21 (30.0) |  | 47 (67.1) | | 23 (32.9) |  |  |  |
| Battambang | 64 (91.4) | | 6 (8.6) |  | 62 (88.6) | | | 8 (11.4) | |  | | 55 (78.6) | | 15 (21.4) |  | 51 (72.9) | | 19 (27.1) |  |  |  |
| Banteay Meanchey | 57 (90.5) | | 6 (9.5) |  | 55 (87.3) | | | 8 (12.7) | |  | | 51 (80.9) | | 12 (19.1) |  | 45 (71.4) | | 18 (28.6) |  |  |  |
| Siem Reap | 65 (89.0) | | 8 (11.0) |  | 58 (79.4) | | | 15 (20.6) | |  | | 51 (69.7) | | 22 (30.1) |  | 46 (63.0) | | 27 (37.0) |  |  |  |
| Kampong Cham | 47 (95.9) | | 2 (4.1) |  | 47 (95.9) | | | 2 (4.1) | |  | | 47 (95.9) | | 2 (4.1) |  | 45 (91.8) | | 4 (8.2) |  |  |  |
| Preah Sihanouk | 47 (88.7) | | 6 (11.3) |  | 43 (81.1) | | | 10 (18.9) | |  | | 37 (69.8) | | 16 (30.2) |  | 34 (64.1) | | 19 (35.9) |  |  |  |
| Age group |  | |  | 0.079 |  | | |  | | 0.254 | |  | |  | 0.480 |  | |  | 0.289 |  |  |
| 18-25 | 276 (89.9) | | 31 (10.1) |  | 261 (85.0) | | | 46 (15.0) | |  | | 200 (65.1) | | 107 (34.9) |  | 188 (61.2) | | 119 (38.8) |  |  |  |
| 26-34 | 232 (85.3) | | 40 (14.7) |  | 222 (81.6) | | | 50 (18.4) | |  | | 17 (61.4) | | 105 (38.6) |  | 149 (54.8) | | 123 (45.2) |  |  |  |
| 35-48 | 62 (93.9) | | 4 (6.1) |  | 51 (77.3) | | | 15 (22.7) | |  | | 45 (68.2) | | 21 (31.8) |  | 38 (57.6) | | 28 (42.4) |  |  |  |
| Marital status |  | |  | 0.007 |  | | |  | | 0.009 | |  | |  | 0.113 |  | |  | 0.070 |  |  |
| Single | 209 (92.9) | | 16 (7.1) |  | 200 (88.9) | | | 25 (11.1) | |  | | 151 (67.1) | | 74 (32.9) |  | 144 (64.0) | | 81 (36.0) |  |  |  |
| Married | 109 (81.9) | | 24 (18.1) |  | 108 (81.2) | | | 25 (18.8) | |  | | 75 (56.4) | | 58 (43.6) |  | 70 (52.6) | | 63 (47.4) |  |  |  |
| Widow/ Separate/ Divorce | 252 (87.8) | | 35 (12.2) |  | 226 (78.7) | | | 61 (21.3) | |  | | 186 (64.8) | | 101 (35.2) |  | 161 (56.1) | | 126 (43.9) |  |  |  |
| Monthly income (X US$100) | 2.6 (1.5) | | 2.7 (1.3) | 0.415 | 2.5 (1.3) | | | 3.1 (2.0) | | 0.002 | | 2.4 (1.2) | | 2.9 (1.7) | <0.001 | 2.4 (1.2) | | 2.9 (1.7) | <0.001 |  |  |
| Number of dependents |  | |  | 0.049 |  | | |  | | 0.198 | |  | |  | 0.523 |  | |  | 0.507 |  |  |
| No dependent | 30 (83.3) | | 6 (16.7) |  | 28 (77.8) | | | 8 (22.2) | |  | | 21 (58.3) | | 15 (41.7) |  | 19 (52.8) | | 17 (47.2) |  |  |  |
| 1 dependent | 87 (95.6) | | 4 (4.4) |  | 81 (89.0) | | | 10 (11.0) | |  | | 64 (70.3) | | 27 (29.7) |  | 59 (64.8) | | 32 (35.2) |  |  |  |
| 2 dependent | 117 (90.0) | | 13 (10.0) |  | 111 (85.4) | | | 19 (14.6) | |  | | 82 (63.0) | | 48 (36.9) |  | 76 (58.5) | | 54 (41.5) |  |  |  |
| 3+ dependent | 336 (86.6) | | 52 (13.4) |  | 314 (80.9) | | | 74 (19.1) | |  | | 245 (63.1) | | 143 (36.9) |  | 221 (57.0) | | 167 (43.0) |  |  |  |
| Childhood family economic status | | |  | 0.039 | | |  | |  | | 0.418 |  | |  | 0.009 |  | |  | 0.010 | |  |
| Poor | | 317 (85.7) | 53 (14.3) |  | | | 306 (82.7) | | 64 (17.3) | |  | 222 (60.0) | | 148 (40.0) |  | 200 (54.0) | | 170 (46.0) |  | |  |
| Medium | | 233 (92.1) | 20 (7.9) |  | | | 212 (83.8) | | 41 (16.2) | |  | 179 (70.7) | | 74 (29.3) |  | 165 (65.2) | | 88 (34.8) |  | |  |
| Rich | | 20 (90.9) | 2 (9.1) |  | | | 16 (72.7) | | 6 (27.3) | |  | 11 (50.0) | | 11 (50.0) |  | 10 (45.4) | | 12 (54.6) |  | |  |
| Education | |  |  | 0.972 | | |  | |  | | 0.424 |  | |  | 0.006 |  | |  | 0.048 | |  |
| No education | | 53 (88.3) | 7 (11.7) |  | | | 47 (78.3) | | 13 (21.7) | |  | 34 (56.7) | | 26 (43.3) |  | 31 (51.7) | | 29 (48.3) |  | |  |
| Primary | | 272 (88.0) | 37 (12.0) |  | | | 251 (81.2) | | 58 (18.8) | |  | 214 (69.3) | | 95 (30.7) |  | 192 (62.1) | | 117 (37.9) |  | |  |
| Secondary | | 192 (89.3) | 23 (10.7) |  | | | 184 (85.6) | | 31 (14.4) | |  | 135 (62.8) | | 80 (37.2) |  | 125 (58.1) | | 90 (41.9) |  | |  |
| Highshcool | | 53 (86.9) | 8 (13.1) |  | | | 52 (85.2) | | 9 (14.8). | |  | 29 (47.5) | | 32 (52.5) |  | 27 (44.3) | | 34 (55.7) |  | |  |
| Living companions | |  |  | 0.009 | | |  | |  | | 0.021 |  | |  | 0.002 |  | |  | 0.006 | |  |
| Alone | | 82 (84.5) | 15 (15.5) |  | | | 71 (73.2) | | 26 (26.8) | |  | 61 (62.9) | | 36 (37.1) |  | 50 (51.5) | | 47 (48.5) |  | |  |
| Relatives | | 197 (89.9) | 22 (10.1) |  | | | 189 (86.3) | | 30 (13.7) | |  | 130 (59.4) | | 89 (40.6) |  | 120 (54.8) | | 99 (45.2) |  | |  |
| Friends | | 204 (92.3) | 17 (7.7) |  | | | 188 (85.1) | | 33 (14.9) | |  | 162 (73.3) | | 59 (26.7) |  | 149 (67.4) | | 72 (32.6) |  | |  |
| Sexual partner/husband | | 87 (80.6) | 21 (19.4) |  | | | 86 (79.6) | | 22 (20.4) | |  | 59 (54.6) | | 49 (45.4) |  | 56 (51.8) | | 52 (48.1) |  | |  |
| Living condition | |  |  | 0.123 | | |  | |  | | 0.016 |  | |  | 0.001 |  | |  | <0.001 | |  |
| Rent a room | | 307 (86.5) | 48 (13.5) |  | | | 279 (78.6) | | 76 (21.4) | |  | 204 (57.5) | | 151 (42.5) |  | 182 (51.3) | | 173 (48.7) |  | |  |
| Own house | | 34 (80.9) | 8 (19.1) |  | | | 36 (85.7) | | 6 (14.3) | |  | 27 (64.3) | | 15 (35.7) |  | 25 (59.5) | | 17 (40.5) |  | |  |
| Friend | | 42 (91.3) | 4 (8.7) |  | | | 42 (91.3) | | 4 (8.7) | |  | 39 (84.8) | | 7 (15.2) |  | 39 (84.8) | | 7 (15.2) |  | |  |
| Relative house | | 46 (93.9) | 3 (6.1) |  | | | 46 (93.9) | | 3 (6.1) | |  | 34 (69.4) | | 15 (30.6) |  | 33 (67.3) | | 16 (32.7) |  | |  |
| Workplace | | 141 (92.2) | 12 (7.8) |  | | | 131 (85.2) | | 22 (14.4) | |  | 108 (70.6) | | 45 (29.4) |  | 96 (62.7) | | 57 (37.3) |  | |  |
|  | |  |  |  | | |  | |  | |  |  | |  |  |  | |  |  | |  |

*FEWs, female entertainment workers; GBV, gender-based violence.*

*Values are the number of subjects (%) for categorical variables and the mean (standard deviation) for continuous variables.*

*^*^Measured by the WHO Multi-Country Study on Women’s Health and Domestic Violence against Women.*

*^†^Chi-square test, or Fisher’s exact test when the sample sizes were smaller than five in one cell, was used for categorical variables. Independent Student’s t-test was used for continuous variables.*
