# Supplementary material for: Gender-based violence and depressive symptoms among female entertainment workers in Cambodia: A cross-sectional study
Source: PLOS Glob Public Health. 2022 Aug 3;2(8):e0000873. doi: 10.1371/journal.pgph.0000873 (PMC10021637; doi:10.1371/journal.pgph.0000873)
Supplement: S2 Table — (DOCX) [file pgph.0000873.s002.docx]

| **Table 2S. Comparison of entertainment and sex work of FEWs with and without experiencing each type of GBV (n=645).** | | | | | | | | | | | | | | | |
| --- | --- | --- | --- | --- | --- | --- | --- | --- | --- | --- | --- | --- | --- | --- | --- |
| **Variable** | **Physical violence*** | | | **Sexual violence*** | | | | **Emotional abuse*** | | | | **At least one type of GBV*** | | |  |
|  | **No** | **Yes** | ***p*-value^†^** | **No** | **Yes** | ***p*-value^†^** | **No** | | **Yes** | ***p*-value^†^** | **No** | | **Yes** | ***p*-value^†^** | |
|  | **N (%)** | **N (%)** |  | **N (%)** | **N (%)** |  | **N (%)** | | **N (%)** |  | **N (%)** | | **N (%)** |  | |
| Duration of entertainment work (<17 months)^‡^ | 375 (88.9) | 47 (11.1) | 0.59 | 348 (82.5) | 74 (17.5) | 0.763 | 271 (64.2) | | 151 (35.8) | 0.804 | 246 (58.3) | | 176 (41.7) | 0.913 | |
| Had borrowed any money in the last 3 months |  |  | 0.004 |  |  | <0.001 |  | |  | <0.001 |  | |  | <0.001 | |
| Yes | 228 (84.1) | 43 (15.9) |  | 207 (76.4) | 64 (23.6) |  | 152 (56.1) | | 119 (43.9) |  | 133 (49.1) | | 138 (50.9) |  | |
| No | 342 (91.4) | 32 (8.6) |  | 327 (87.4) | 47 (12.6) |  | 260 (69.5) | | 114 (30.5) |  | 242 (64.7) | | 132 (35.3) |  | |
| Type of current working place |  |  | 0.299 |  |  | <0.001 |  | |  | <0.001 |  | |  | <0.001 | |
| Karaoke | 240 (86.3) | 38 (13.7) |  | 228 (82.0) | 50 (18.0) |  | 180 (64.7) | | 98 (35.3) |  | 163 (58.6) | | 115 (41.4) |  | |
| Massage | 101 (92.7) | 8 (7.3) |  | 92 (84.4) | 17 (15.6) |  | 85 (78.0) | | 24 (22.0) |  | 79 (72.5) | | 30 (27.5) |  | |
| Beer Garden | 23 (88.5) | 3 (11.5) |  | 24 (92.3) | 2 (7.7) |  | 15 (57.7) | | 11 (42.3) |  | 14 (53.8) | | 12 (46.2) |  | |
| Restaurant | 108 (90.8) | 11 (9.3) |  | 110 (92.4) | 9 (7.6) |  | 77 (64.7) | | 42 (35.3) |  | 71 (59.7) | | 48 (40.3) |  | |
| Dance Club | 72 (87.8) | 10 (12.2) |  | 56 (68.3) | 26 (31.7) |  | 34 (41.5) | | 48 (58.5) |  | 29 (35.4) | | 53 (64.6) |  | |
| Street | 7 (70.0) | 3 (30.0) |  | 6 (60.0) | 4 (40.0) |  | 5 (50.0) | | 5 (50.0) |  | 4 (40.0) | | 6 (60.0) |  | |
| Bar | 19 (90.5) | 2 (9.5) |  | 18 (85.7) | 3 (14.3) |  | 16 (76.2) | | 5 (23.8) |  | 15 (71.4) | | 6 (28.6) |  | |
| Satisfaction with current job |  |  | 0.049 |  |  | 0.063 |  | |  | 0.047* |  | |  | 0.030* | |
| Unsatisfied | 17 (80.9) | 4 (19.1) |  | 12 (57.1) | 9 (42.9) |  | 7 (33.3) | | 14 (66.7) |  | 5 (23.8) | | 16 (76.2) |  | |
| Somewhat satisfied | 118 (82.5) | 25 (17.5) |  | 118 (82.5) | 25 (17.5) |  | 89 (62.2) | | 54 (37.8) |  | 83 (58.0) | | 60 (42.0) |  | |
| Neutral | 22 (84.6) | 4 (15.4) |  | 22 (84.6) | 4 (15.4) |  | 18 (69.2) | | 8 (30.8) |  | 15 (57.7) | | 11 (42.3) |  | |
| Mostly satisfied | 361 (90.5) | 38 (9.5) |  | 336 (84.2) | 63 (15.8) |  | 260 (65.2) | | 139 (34.8) |  | 239 (59.9) | | 160 (40.1) |  | |
| Very satisfied | 52 (92.9) | 4 (7.1) |  | 46 (82.1) | 10 (17.9) |  | 38 (67.9) | | 18 (32.1) |  | 33 (58.9) | | 23 (41.1) |  | |
| Ever been forced to drink |  |  | <0.001 |  |  | <0.001 |  | |  | <0.001 |  | |  | <0.001 | |
| No | 498 (91.0) | 49 (9.0) |  | 473 (86.5) | 74 (13.5) |  | 379 (69.3) | | 168 (30.7) |  | 349 (63.8) | | 198 (36.2) |  | |
| Yes | 72 (73.5) | 26 (26.5) |  | 61 (62.2) | 37 (37.8) |  | 33 (33.7) | | 65 (66.3) |  | 26 (26.5) | | 72 (73.5) |  | |
| Engage in transactional sex |  |  | 0.001 |  |  | <0.001 |  | |  | 0.001 |  | |  | <0.001 | |
| No | 355 (91.7) | 32 (8.3) |  | 358 (92.5) | 29 (7.5) |  | 267 (69.0) | | 120 (31.0) |  | 257 (66.4) | | 130 (33.6) |  | |
| Yes | 215 (83.3) | 43 (16.7) |  | 176 (68.2) | 82 (31.8) |  | 145 (56.2) | | 113 (43.8) |  | 118 (45.7) | | 140 (54.3) |  | |
|  |  |  |  |  |  |  |  | |  |  |  | |  |  | |

*FEWs, female entertainment workers; GBV, gender-based violence.*

*Values are the number of subjects (%) for categorical variables and the mean (standard deviation) for continuous variables.*

*^*^Measured by the WHO Multi-Country Study on Women’s Health and Domestic Violence against Women.*

*^†^Chi-square test, or Fisher’s exact test when the sample sizes were smaller than five in one cell, was used for categorical variables. Independent Student’s t-test was used for continuous variables.*
