# Supplementary material for: Gender-based violence and depressive symptoms among female entertainment workers in Cambodia: A cross-sectional study
Source: PLOS Glob Public Health. 2022 Aug 3;2(8):e0000873. doi: 10.1371/journal.pgph.0000873 (PMC10021637; doi:10.1371/journal.pgph.0000873)
Supplement: Attachment — Submitted filename: Response to Reviewers.docx [file pgph.0000873.s004.docx]

Responses to Reviewers

Reviewer #1

|  | Reviewer’s comment | Author’s response |
| --- | --- | --- |
| Introduction | | |
| 1 | Lines 49-50: "In particular, female sex workers (FSWs), who often come from poor and rural families, are at higher risk of GBV (3)." I think you need to add "in Cambodia" to this sentence. This is not generalisable at the global level. | Thank you very much for your reviews and comments. We have addressed your comments below:  In this paragraph, we would like to introduce the global background of GBV among female sex workers (FSWs). However, many existing studies focused on violence among FSWs who are similar but somehow different from female entertainment workers (FEWs) in the Cambodian context. This sentence was based on a systematic review of the correlates of violence against sex workers from 39 countries. We have clarified the sentence as you suggested on lines 57-59:  *According to a systematic review of the correlates of violence against sex workers in 39 countries, female sex workers (FSWs), who often come from poor and rural families, are at high risk of GBV (3).*  We also introduced the background of GBV among FEWs in Cambodia in paragraph 5, lines 83-88:  *In Cambodia,* *FEWs experience high levels and multiple forms of GBV confronted with different perpetrators, including intimate partners, clients, and entertainment establishment owners or managers (19). Two-thirds of FEWs reported having experienced GBV, including emotional abuse, physical abuse, forced sex, and forced substance use in their lifetime (19). FEWs, especially those who engage in transactional sex, face considerable risks to GBV, including rape and gang rape, by clients (13).* |
| Methods | | |
| 2 | Line 123: Please define KHANA | We have addressed this in the manuscript on lines 131-133:  *First, we obtained a list of venues in the seven study sites* *from the program team of Khmer HIV/AIDS NGO Alliance (KHANA), a local non-government organization.* |
| 3 | Lines 172-175: Were these thresholds based on previous research? Please explain. | The thresholds were based on a validation study, which has shown that a cutoff score of 16 aids in identifying individuals at risk for clinical depression, with good sensitivity and specificity and high internal consistency.  We have added this information on lines 185-186:  *This threshold aids in identifying individuals at risk for clinical depression with good sensitivity and specificity and high internal consistency (31).* |
| 4 | Lines 178-179: The WHO study is related to Intimate Partner Violence - has it been adapted to allow measurement of non-partner violence in the context of this questionnaire? Or did the researchers include the WHO non-partner measure? More detail is required on the measurement tools. | This questionnaire contained questions that capture specific acts of each form of violence, including emotional, physical, and sexual violence. This tool produced a reliable and valid measurement of women’s experiences of violence since it explicitly operationalizes and provides clear definitions of specific acts of violence. This tool also gives multiple opportunities to respondents to assess whether their experiences fit within the objectives of the survey and the specific questions being asked to provide more detailed accounts of their experiences. Therefore, we adapted the questionnaire to measure non-partner violence following the Guidelines for Producing Statistics on Violence against Women. For example, FEWs were asked, “In the past 12 months, has anyone ever hit, beaten, slapped, kicked, pulled you by the hair, or thrown something at you to hurt you?”  Moreover, we conducted the initial meeting with FEWs before the study and added a few questions to capture all forms of violence in the context of FEWs in Cambodia. We have added more details of the tool as suggested on lines 190-192:  *We adapted a questionnaire from the WHO Multi-Country Study on Women’s Health and Domestic Violence against Women, following the Guidelines for Producing Statistics on Violence against Women (27,32).*  We have also added more details of the questionnaire on lines 193-199:  *We asked FEWs about their experiences in different forms of GBV in the past 12 months, including physical violence (ever been hit or thrown something, been threatened with gun or weapon, been choked or burned on purpose), sexual violence (been forced to do something sexually, not been paid for sex or paid less, been forced to do something sexual, been forced to engage in group sex), and emotional abuse (been yelled at or insulted, been intimidated or scared and threatened with harm).* |
| 5 | Lines 181-183: The measure is described as "past 12 months" (line 181) and "ever" (line 183), it can not be both. Please clarify. | The questionnaire asked FEWs if they have experienced any forms of physical violence, sexual violence, and emotional abuse in their lifetime and the last 12 months. However, in this analysis, we included only their experiences in the past 12 months. We have clarified this on lines 199-200:  *We dichotomized the response into yes or no to determine the proportions of ever experiencing at least one form of GBV described above in the past 12 months.* |
| 6 | Line 223-230: While the researchers describe the ethical considerations in this section, there is limited description of safety considerations for the participants. I find this concerning when it appears that the study was undertaken with the knowledge of entertainment venue managers and managers may be perpetrators of violence. I think there needs to be a more comprehensive description of the methods of recruitment. In particular, how were participants recruited without the knowledge of managers? If this wasn't possible, what considerations were made for their safety? How was the study described to the entertainment venue managers? | The study was conducted as part of a large-scale community-based HIV and GBV project among FEWs under the national HIV program. The project involved local health authorities, community-based organizations, and entertainment owners/managers. We have added more information about recruitment on lines 154-156:  *Next, the data collectors contacted the selected FEWs by telephone. Then, data collectors screened the selected FEWs for eligibility with a screening tool and made an appointment with them for a face-to-face interview.*  Before conducting the interviews, data collectors explained to FEWs about the study. They provided the information for access to free counseling services and relevant referrals in case FEWs wanted to speak to someone during or after the research study. We have also added this information in the ethical consideration section on lines 244-245:  *Data collectors also provided information about free counseling and relevant referral services in case FEWs wanted to speak to someone during or after the study.* |
| Results | | |
| 7 | Table 2: Please describe the comparisons for the p-values. For example, the bottom 2 rows appear to contain only those who answered "yes" to each of these items. Are they compared with the "no" responses? Please provide results for the "no" responses if these are the comparison groups. | In Table 2, *p*-values were obtained from the Chi-square test of each variable with depressive symptoms. Thus, the p-values indicate a significant difference in the distribution of each variable between high and low levels of depressive symptoms. Per your suggestion, we have modified Table 2 by adding the “No” responses to binary variables. |
| Discussion | | |
| 8 | Line 319-320: This sentence needs to be rewritten. | Per your suggestion, we have changed the sentence on lines 344-45:  *Among three types of GBV, emotional abuse was the most experienced by FEWs in this study. Emotional abuse was also associated with depressive symptoms among FEWs.* |
| 9 | Page 21: I think this whole page is underscoring how difficult it can be to disentangle the effects of physical/sexual or emotional violence on depressive symptoms. While I think this is what the researchers are explaining, I think it would be useful to make it more explicit - "While we found no independent association between physical or sexual violence and depressive symptoms, the high degree of overlap between physical, sexual and emotional violence may make the identification of an independent association difficult in the current investigation" (or something like this). If the variables were all added at the same time to the model, it is possible that the emotional violence measure is just accounting for the variation in the physical or sexual violence measures also. | Per your suggestion, we have added a more explicit paragraph on lines 383-386:  *While we found no independent association between physical or sexual violence and depressive symptoms, the high degree of overlap between physical, sexual, and emotional violence may make identifying an independent association complex in the current investigation.* |

Reviewer #2

|  | Reviewer’s comment | Author’s response |
| --- | --- | --- |
| Method | | |
| 1 | According to the methodology, 10 percent of entertainment venues were chosen from each location, and a maximum of seven FEWs were chosen from each entertainment facility. However, it appears that one site, Phnom Penh, has more than 10 percent of the total venues and hence represents a more proportion of FEWs in the study (n = 267, nearly 41 percent). To better understand the variances in the selection process, additional clarity is required on this point. | Thank you very much for your reviews and comments. We have addressed your comments below:  During the sampling process, we chose 10 percent of the total entertainment venues available at each site to make the sample size representative. This number would be higher for sites with a higher total number of entertainment venues. As a result, the highest number of entertainment venues were chosen from Phnom Penh because Phnom Penh had the highest total number of entertainment venues compared to other locations.  Per your suggestion, we have clarified it on lines 135-136:  *Second, we randomly selected 10% of the total number of entertainment venues in each study site.* |
| Analysis | | |
| 2 | Because the GVB is one of the primary study indicators, analysing GBV by background profile may provide readers with a better understanding. | Per your suggestion, we have performed additional analyses of GBV by background profile and showed the results in tables 1S and 2S (see supplementary document). |
| Results and Discussion | | |
| 3 | The majority of the FSWs were mostly (n=399) and satisfied (n=56) with their current work; only a few were unsatisfied (n=21). Some discussion or reflection would be beneficial for women in abusive workplaces with/without mental health conditions to rate the satisfaction of their current employment. | As you suggested, we have added more discussion about the satisfaction of current employment among FEWs on lines 397-404:  In this study, most of the FEWs were satisfied with their current work. This could be because they accept certain aspects of their work, including tips, dancing, singing, and feeling cared for by the entertainment establishment owners (16). A previous study also showed that FEWs enjoyed their work compared to potential alternative choices, such as moving back to their village and cultivating rice like their parents (16). Nevertheless, this result might not imply that the working environment in entertainment venues was favorable. Some FEWs were unsatisfied with their job, which might be due to the long working hours, the nature of the work, and their status in society (16). |
| 4 | Some thoughts on how current policy fails to protect FEWs from GBV and fails to address their mental health needs. | Per your suggestion, we have added more information about previous policies in Cambodia and some recommendations on lines 405-413:  Previous programs in Cambodia, such as the NGO-led SMARTGirl, provided a support network for GBV survivors (13). However, these programs do not necessarily contain legal support to prevent GBV and emotional support from health professionals (13). Moreover, FEWs often face difficulties accessing services due to societal stigmatization (16). Addressing GBV and mental health problems among FEWs requires a multifaceted approach. These could be done by providing legal and emotional support that FEWs can easily access, promoting and raising awareness of GBV and mental health among FEWs, and involving entertainment establishment owners and managers in innovative interventions to address emotional, physical, and sexual violence. |
